# THE MEDIATING EFFECT OF SELF-OBJECTIFICATION AND BODY ESTEEM ON AGE AND MOOD

**DOI:** 10.1093/geroni/igac059.2403

**Published:** 2022-12-20

**Authors:** Sydney Tran, Aurora Sherman

**Affiliations:** Oregon State University, Corvallis, Oregon, United States; Oregon State University, Corvallis, Oregon, United States

## Abstract

Objectification theory is a well-established framework that outlines the consequences of being sexually objectified (Fredrickson & Roberts, 1997). One of the major consequences of sexual objectification is self-objectification, or the tendency to internalize an observer’s perspective on one’s own body and the learned behavior to value one’s physical appearance over one’s body functionality. Self-objectification has been linked to poorer subjective well-being and poorer body image in college-aged women but has not yet been examined among older adult women. This study recruited a group of younger (N = 132 ; M age = 20.93, range = 18-26) and older (N = 86; M age = 67.83, range = 48-90) adult women to examine age differences in self-objectification and their relationships with body esteem and negative mood and anxiety. This study tested a serial mediation model using Model 6 of the Hayes PROCESS Macro in SPSS, in which self-objectification and body esteem were hypothesized to mediate the relationship between age and negative mood and anxiety. After controlling for sexual orientation, marital status, and education, results indicated that the standardized indirect effect was significant, B = -.09, SE = .03, 95% CI [-.16, -.04], suggesting that self-objectification and its related consequences are not unique to young adult women and that women of all ages are negatively impacted by self-objectification.

